# CD19 CAR-T Outcomes in Patients with Relapsed/Refractory Diffuse Large B-Cell Lymphoma: A Retrospective Cohort Study from the Calabria Referral Center in Southern Italy

**DOI:** 10.3390/cancers17172796

**Published:** 2025-08-27

**Authors:** Daniele Caracciolo, Filippo Antonio Canale, Virginia Naso, Caterina Alati, Violetta Marafioti, Gaetana Porto, Ludovica Tedesco, Giulia Pensabene, Enrica Antonia Martino, Alessandro Allegra, Demetrio Gabriele Gerace, Michele Cimminiello, Massimo Gentile, Pierosandro Tagliaferri, Pierfrancesco Tassone, Massimo Martino

**Affiliations:** 1 Department of Experimental and Clinical Medicine (DMSC), Magna Graecia University, 88100 Catanzaro, Italy; tagliaferri@unicz.it (P.T.); tassone@unicz.it (P.T.); 2 Medical and Translational Medical Oncology Units, Phase 1 Trial Center in Oncology and Onco-Hematology, AOU Renato Dulbecco, 88100 Catanzaro, Italy; ludovica.tedesco@studenti.unicz.it (L.T.); giulia.pensabene@studenti.unicz.it (G.P.); 3 Hematology and Stem Cell Transplantation and Cellular Therapies Unit (CTMO), Department of Hemato-Oncology and Radiotherapy, Grande Ospedale Metropolitano “Bianchi-Melacrino-Morelli”, 89133 Reggio Calabria, Italy; filippo.canale@ospedalerc.it (F.A.C.); virginia.naso@ospedalerc.it (V.N.); caterina.alati@ospedalerc.it (C.A.); violetta.marafioti@ospedalerc.it (V.M.); gaetana.porto@ospedalerc.it (G.P.); 4 Hematology Unit, Department of Onco-Hematology, Azienda Ospedaliera Annunziata, 87100 Cosenza, Italy; ea.martino@aocs.it (E.A.M.); massimo.gentile@unical.it (M.G.); 5 Division of Hematology, Department of Human Pathology in Adulthood and Childhood “Gaetano Barresi”, University of Messina, 98124 Messina, Italy; aallegra@unime.it (A.A.); demetriogabriele.gerace@polime.it (D.G.G.); 6 Hematology and Stem Cell Transplant Unit, Azienda Ospedaliera San Carlo, 85100 Potenza, Italy; 7 Department of Pharmacy, Health and Nutritional Science, University of Calabria, 87036 Rende, Italy

**Keywords:** CAR-T, hematologic malignancies, DLBCL, lymphoma, immunotherapy

## Abstract

Background: Chimeric antigen receptor (CAR) T-cell therapy has transformed the therapeutic landscape for relapsed or refractory (R/R) diffuse large B-cell lymphoma (DLBCL). Our study aims to describe the clinical outcomes of CAR T-cell therapy in patients with R/R DLBCL treated at a single regional center in Italy, with the goal of comparing these outcomes to those reported by high-volume academic centers. Methods: Data were retrospectively collected from a cohort of consecutive 41 patients who underwent to CD19 CAR-T infusion from June 2020 until September 2024 at CAR-T center of Reggio Calabria (Italy). Results: The median age was 66 years, 60.9% were refractory to their most recent regimen, and 24.4% had previously failed autologous stem cell transplant. Bridging therapy was administered in 82.9% of cases. A total of 27 patients (65.8%) received Axi-cel, and 14 (34.2%) received Tisa-cel. At median follow-up of 6.9 months, the best ORR and CR rate were 63.4% and 51.2%, respectively. Median PFS was 3 months, and median OS was 8.4 months. A total of 81.4% of patients developed a CRS, grade 1 in most cases (78.4%); 26.8% developed ICANS: two (5.4%) and three (8.1%) had grade 2 and 3, respectively. In univariate analyses, early response predicted longer survival, whereas high tumor burden and more than one extranodal site were associated with worse outcomes. Conclusions: Our retrospective cohort study reports similar data in terms of clinical response as compared to pivotal trials and other reports, confirming that CAR-T may offer more durable response rates and longer progression-free intervals in R/R DLBCL in our real-world context.

## Simple Summary

We report our experience at a regional CAR-T center in southern Italy, where 41 patients with relapsed or refractory large B-cell lymphoma (DLBCL) were treated with two approved CAR-T products, Axi-cel and Tisa-cel. By analyzing the outcomes, safety, and treatment pathways of these patients, this study aims to evaluate how effective and feasible these advanced therapies are in a real-world setting. The findings not only confirm the clinical benefit of CAR-T therapy in this population as compared to pivotal trials but also highlight the importance of early patient referral, appropriate selection, and strong supportive care. These insights can help improve treatment strategies and support health system decisions about the use of CAR-T therapy in routine practice.

## 1. Introduction

Diffuse large B-cell lymphoma (DLBCL) is the most common type of non-Hodgkin’s lymphoma (NHL) and can be cured by standard immunochemotherapy (R-CHOP) in about 60% of cases [1,2]. However, after first-line therapy, 30–40% of patients finally relapse, with an additional 10% showing primary refractory disease [3]. In these clinical settings, until recently, the standard therapeutic option was salvage high-dose therapy (HDT) followed by autologous stem cell transplantation (ASCT) in chemo-sensitive patients [4]. Unfortunately, patients who are not eligible for ASCT because of being refractory to salvage therapy or for comorbidities or due to relapse occurring within 12 months after ASCT have poor outcome [5,6].

Second-generation CD-19 CAR-T cells axicabtageneciloleucel (Axi-cel) and tisangenleucel (Tisa-cel) [7] are approved by the Italian Medicines Agency (AIFA) for R/R DLBCL on the basis of single-arm phase II ZUMA-1 [8] and JULIET trials [9]. Indeed, in patients with DLBCL failing two or more lines of treatment, overall response rates (ORRs) ranging from 52% to 83% with complete response rate (CRRs) of 40% to 58% were reported. More recently, ZUMA-7 trial showed superior event-free survival [10], response rate, and overall survival (OS) versus standard care practices [11] in transplant-intended R/R DLBCL. These data led to Axi-cel approval in 2023 by AIFA as second-line treatment for patients refractory to first-line treatment or that had relapsed within 12 months after first-line chemoimmunotherapy.

Stringent eligibility criteria of pivotal clinical trials often restrict the applicability of the outcomes in real-world populations. In fact, in this setting, patients are typically older, more heavily pretreated, and present with a higher burden of comorbidities or disease-related complications that would have excluded them from trial participation.

Real-world studies are therefore essential to assess the true feasibility, safety, and effectiveness of CAR-T therapies outside the controlled environment of clinical trials [12]. These studies help clarify treatment outcomes in more heterogeneous and clinically complex populations, identify potential logistical or systemic barriers (e.g., access delays, manufacturing timelines), and contribute to the optimization of patient selection, toxicity management, and supportive strategies, especially in non-centralized healthcare settings [13].

This retrospective real-world cohort study aims to describe the clinical outcomes and safety profile of both approved CD19-targeted CAR-T-cell therapies administered as standard of care to patients with DLBCL treated at a single regional center in Italy, with the goal of comparing these outcomes to those reported by high-volume academic centers.

## 2. Patients and Methods

Data from all consecutive patients with R/R DLBCL who underwent leukapheresis at CAR-T center of Reggio Calabria (Calabria, Italy), from June 2020 until September 2024, were retrospectively collected and described in accordance with methodological quality criteria for retrospective cohort studies, as outlined in the STROBE checklist (version 4) [14].

All patients who received CD-19 CAR-T-cell infusion and had at least a post-infusion restaging (at 1 month) were included for efficacy and safety analysis.

In accordance with Agenzia Italiana del Farmaco (AIFA) reimbursement guideline, this study’s eligibility criteria were age between 18 and 75 years; Eastern Cooperative Oncology Group (ECOG) performance status of 0–1; normal organ function; progression after at least one prior line of therapy; diagnosis of DLBCL or DLBCL transformed from follicular, marginal zone, or Hodgkin’s lymphoma (tFL, tMZL, tHL). All patients underwent to screening procedures, including laboratory blood tests, echocardiography, and positron emission tomography/computed tomography (PET/CT) scans.

CAR-T product (Axi-cel or Tisa-cel) infusion choice was dependent only on slot production availability and specific approval setting. In particular, patients with DLBCL who relapsed after 2 lines of therapy (from third line) were treated with Tisa-cel, which was the first CAR-T product to be approved by AIFA (7 August 2019), followed by Axi-cel (11 November 2019) (Supplementary Table S1). For this reason, the first 14 patients of our cohort were consecutively treated with Tisa-cel because was the only product approved at that time. In contrast, for patients at their first DLBCL relapse (from second line), only Axi-cel was used, as it received specific AIFA approval in November 2023, while Tisa-cel did not due the BELINDA trial results [15].

Bridging therapy was allowed as part of the treatment plan. Fludarabine and cyclophosphamide (FC) were administered to all included patients as lymphodepleting chemotherapy (LC) [16]. CAR-T cells were infused in an inpatient setting to allow strict monitoring and management of potential adverse events (AEs). Hospitalization was maintained for a minimum of 14 days post-infusion, while clinical follow-up was conducted on an outpatient basis. For efficacy outcomes, all patients underwent a baseline PET/CT scan, repeated 1, 3, 6, 12, 18, and 24 months after infusion, for the assessment of response according Lugano’s recommendations [17].

Efficacy endpoints of the study were the overall response rate (ORR), overall survival (OS), and progression-free survival (PFS), as previously defined [18]. For the safety profile endpoint, cytokine release syndrome (CRS) and immune effector cell-associated neurotoxicity syndrome (ICANS) were graded according to the American Society for Transplantation and Cellular Therapy (ASCT) criteria [19]. For the recording of other AEs, Common Terminology Criteria for AEs (CTCAE) version 6.0 was used (https://dctd.cancer.gov/research/ctep-trials/trial-development) (accessed on 9 March 2025). The median follow-up was calculated from the date of infusion to January 2025, the most recent update date. Baseline characteristics of study population are reported using descriptive statistics. Survival outcomes were estimated using the Kaplan–Meier method. Univariable Cox regression model was used to identify variables suspected of independently correlating to OS or toxicity (CRS or ICANS). Variables with a *p*-value ≤ 0.1 were considered for multivariable analysis using the Cox regression model, calculated with the Wald backward method.

All statistical analyses [20,21] were conducted using SPSS version 25.0 (IBM Corp., Chicago, IL, USA), with a significance level set at *p* < 0.05.

## 3. Results

### 3.1. Baseline Population Characteristics

From September 2020 to September 2024, 47 patients with DLBCL (three tFL, two tHL, and two tMZL) underwent leukapheresis at our CAR-T center. Of these, six patients were excluded by our analysis for incomplete information during follow-up: three for early death due to progressive disease (PD) (*n* = 2) or COVID-19 (*n* = 1); two for not-allowed histology; one due to missing data. Subsequently, 41 eligible patients received CAR-T-cell infusion, without significant issues in shipping leukapheresis samples or receipt of CAR-T product. In particular, 27 (65.8%) received Axi-cel (10 in second line, 17 from third line of treatment), while 14 (34.2%) were treated with Tisa-cel (from third line), with a median time from apheresis to infusion (vein-to-vein, V2Vt) of 28.5 days (IQR 23–42). Baseline demographic and clinical characteristics are detailed in Table 1. In summary, median age at the time of leukapheresis was 66 years (IQR 52–68.5), and 32 patients (78%) were men. Most were refractory to last treatment (60.9%) and had bulky disease (56.7%) at apheresis. Only 4.9% had performance status ECOG > 1. All patients had received multiple prior lines of therapies, with a median number of 2 (range 1–4). Prior autologous stem cell transplantation (ASCT) had been performed in 10 patients (24.4%). Bridging therapy prior to infusion was administered in 34 patients (82.9%), including 48.7% chemoimmunotherapy, 19.5% chemo alone, 2.5% lenalidomide, 7.3% radiotherapy, 7.3% steroids alone. In 17.1% of cases no bridging therapy was performed.

### 3.2. Efficacy and Outcomes

**Figure 1:**
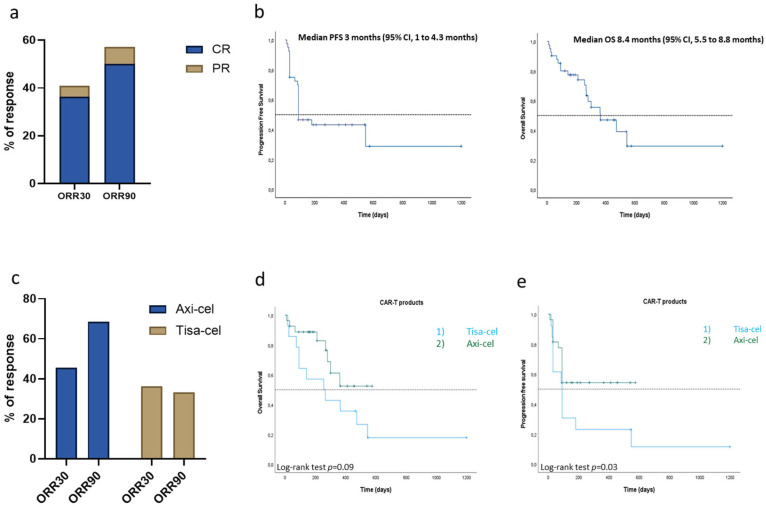
Efficacy in overall population and after stratification by CAR-T product infused. (**a**) ORR (with % of CR and PR) at day 30 and 90 post-CAR-T infusion. (**b**) OS and PFS in overall cohort with DLBCL treated with CAR-T cells. (**c**) ORR at day 30 and 90 post-Axi-cel and -Tisa-cel infusion. (**d**,**e**) Kaplan–Meier curves for OS (**d**) and PFS (**e**) comparing Axi-cel- versus Tisa-cel-treated patients. Significance level set at *p* < 0.05.

At median follow-up of 6.9 months (IQR: 3.4–12.7), the best ORR and CR were 63.4% and 51.2%, respectively. At 30 days post-infusion, the ORR (ORR30) was 40.9% (36.4% CR and 4.5% PR), while at 90 days post-infusion, the ORR (ORR90) was 57.1% (50% CR and 7.1% PR) (Figure 1a). From the time of CAR-T infusion, median PFS was 3 months (95% CI, 1 to 4.3 months), and median OS was 8.4 months (95% CI, 5.5 to 8.8 months) (Figure 1b).

When stratified by CAR-T product (Table 2), ORR30 was 45.5% for Axi-cel (36.3% CR) and 36.7% for Tisa-cel (33.4% CR) (*p* = 0.5), while ORR90 was 68.4% (63.1% CR) for Axi-cel and 33.3% (22.2% of CR) for Tisa-cel (*p* = 0.08) (Figure 1c). Furthermore, median OS was 8.4 months for Tisa-cel (95% CI, 0.9 months to 15.85 months) and was not reached for Axi-cel (*p* = 0.09), while median PFS was 3 months for Tisa-cel (95% CI, 1.34 months to 4.56) and was not reached for Axi-cel (*p* = 0.04) (Figure 1d,e).

**Figure 2:**
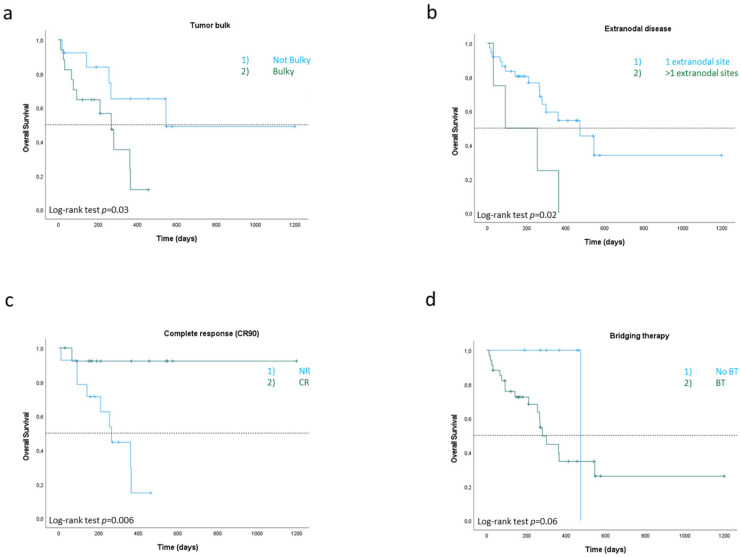
Subgroup analysis of overall survival. Kaplan–Meier curve for OS of all treated patients based on bulky disease (**a**), number of extranodal sites (**b**), complete response rate at 90 days post-infusion (**c**), and bridging therapy to CAR-T (**d**). Significance level set at *p* < 0.05.

Stratifying by disease involvement at diagnosis, patients with bulky disease and >1 of extranodal sites showed poor prognosis. Indeed, median PFS was 18 months in patients without bulky disease (95% CI, 17.7 to 18.1 months), compared to a median PFS of 3 months (95% CI, 2.2 to 3.7 months) in patients with tumor bulk (*p* = 0.01). Similarly, median OS was 17.8 months in patients without bulky disease (95% CI, 17.5 to 18 months), compared to a median OS of 8.7 months (95% CI, 6 to 11.6 months) in patients with bulky disease (*p* = 0.03). In the same way, DLBCL patients with >1 extranodal sites had a median OS of 3 months (95% CI, 2.2 to 11 months), compared to a median OS of 15.5 months (95% CI, 8.2 to 22.8 months) in those with only one extranodal site (Figure 2a,b).

Among patients who achieved CR to CAR-T-cell therapy, median PFS was 18 months (95% CI, 3.45 to 32.4 months) compared to a median PFS of 3 months (95% CI, 2.8 to 3.1 months) in patients who did not achieve a CR (*p* = 0.01). Similarly, median OS was not reached in patients who achieved a CR compared to a median OS of 8.7 months (95% CI, 5.85 to 11.56 months) in patients who did not achieve a CR (*p* = 0.006) (Figure 2c).

No significant difference in CR rate was observed between patients who were administered bridging therapy and those who were not (*p* = 0.49). In the non-bridging cohort, median PFS was not reached, whereas a median PFS of 3.0 months (95% CI, 1.57–4.35) was observed in patients who underwent bridging therapy (*p* = 0.36). A median OS of 9.1 months (95% CI, 7.59–10.73) was reported in the bridging group, while it was not reached in the cohort without bridging therapy (*p* = 0.06) (Figure 2d).

Our analysis included also a small cohort (*n* = 10) of patients with DLBCL treated at first relapse. At the time of this study, only Axi-cel had received AIFA approval in this setting. Despite the small patient number, when stratifying by line of treatment, median PFS was not reached in patients treated in the second line (Axi-cel) and was 3 months (95% CI, 2.8 months to 3.1) (*p* = 0.174) in patients infused from third line. Median OS was not reached in patients treated in second line (Axi-cel) and was 1 year (95% CI, 5.8 months to 12) (*p* = 0.834) from third line of treatment (Supplementary Figure S1).

Upon univariate analysis, ORR at 30 and 90 days was significantly associated with better prognosis, while tumor bulk and >1 extranodal sites correlated with worse outcomes. Cox regression analysis was performed to determine the clinical risk factors for OS and included covariates with a *p*-value  ≤  0.1 from the univariate analysis.

After adjustment using multivariate Cox regression analysis, no statistically significant associations were observed between most of the evaluated factors and the outcomes of interest in our cohort (Table 3, Supplementary Table S2).

### 3.3. Safety

In patients who received Axi-cel, eight died (29.6%): three deaths were related to lymphoma relapse/progression, one to COVID-19, two for CRS, two for non-relapse events, and two were unknown. In patients who received Tisa-cel, 78.5% died: sox deaths were related to lymphoma relapse/progression, two for COVID-19, one was due to non-relapse mortality, and four were unknown.

**Figure 3:**
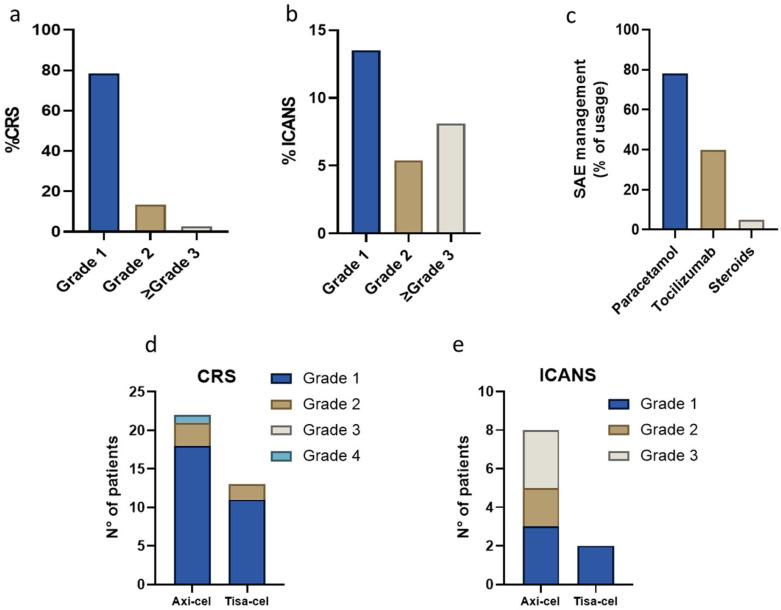
Safety analysis in overall population and by CAR-T product infused. (**a**,**b**) CRS (**a**) and ICANS (**b**) onset and severity in treated patients. (**c**) Management modality of CAR-T’s side effects. (**d**,**e**) CRS (**d**) and ICANS (**e**) onset, based on CAR-T product infused.

The majority of CRS (Figure 3a) and ICANS (Figure 3b) observed in our cohort were grade 1 and 2. In particular, CRS of any grade was observed in 35 patients (81.4%) and in most cases it was of grade 1 (78.4%). ICANS of any grade was observed in 11 patients (26.8%), with grade 2 and 3 ICANS reported in two (5.4%) and three (8.1%) patients, respectively (Table 4). The median time to onset was 2 days for CRS (range 0–11), including three cases occurring on the same day as the infusion, and 5 days for ICANS (range 3–13). A single case of macrophage activation syndrome (MAS) was reported and managed with high-dose corticosteroids, resulting in partial clinical improvement.

In our cohort, only bulky tumor was associated with increased risk of toxicity (CRS or ICANS) (Supplementary Tables S3 and S4).

Management of CAR-T-related adverse events (AEs) required paracetamol in 77.1% of patients, while 65% were treated with tocilizumab (1–3 administered doses). Among these patients, 5.1% were additionally treated with corticosteroids due to refractoriness to tocilizumab (Figure 3c). Admission to the intensive care unit (ICU) was not required for any patients included in this study (Table 4).

No one had a life-threatening infectious disease, except for COVID-19, which was the cause of death in three patients. At 30 days post-infusion, 22% of patients had cytopenia, mainly represented by neutropenia (ANC < 1000/mm^3^) (26.8%).

By stratifying patients by CAR-T product, no difference was observed in terms of CRS or ICANS overall incidence, but grade 3 or higher CRS and ICANS occurred in 3.7% and in 11.1% of Axi-cel treated patients, which was not observed in Tisa-cel group (*p* = 0.09) (Figure 3d,e).

## 4. Discussion

The present real-world analysis reports the clinical outcomes and safety profile of CD19-directed CAR-T-cell therapy in 41 patients with R/R DLBCL treated at a regional center in southern Italy between September 2020 and September 2024. Our cohort is representative of the heterogeneity and complexity of this subgroup of patients, offering valuable insights into the effectiveness and safety of CAR-T therapy in routine clinical practice, either as second-line treatment (Axi-cel) or after two or more lines of therapy (Axi-cel and Tisa-cel). Indeed, it is important to note that over two-thirds of our cohort were ineligible for ZUMA-1 and ZUMA-7, primarily due to receipt of bridging therapy. Furthermore, the median age of our population was significantly greater than that in pivotal clinical trials, with a median age of 66 years as compared to a median age of 58 years in ZUMA-1 and ZUMA-7 and 56 years in JULIET trials (9–11).

Despite our short follow-up (6.9 months), which requires longer observation for more definitive conclusions, our real-world study found an ORR of 63.4% and a CR of 51.2%, which are slightly lower than those reported in ZUMA-1 (ORR, 82%; CR, 54%) but quite similar to the data from the JULIET trial (ORR, 52%; CR, 40%). Of relevance, our study also included a small cohort of patients with R/R DLBCL treated with CAR-T at the first relapse, showing impressive results, with an ORR of 75% (62% CR); median PFS and OS were not reached, with data almost comparable to those of largest pivotal trial, ZUMA-7 [ORR 83% (CR, 65%), median PFS 8.3 months, and median OS not reached].

Importantly, our data are consistent with the growing body of real-world experience from larger U.S. and European CAR-T centers, which demonstrate the feasibility, efficacy, and reasonable toxicity profile of such therapeutic approaches in peripheral non-academic contexts [22,23,24,25,26].

In particular, clinical benefit was clear in patients achieving a complete response (CR), who had a median PFS of 18 months, and a median OS was not reached. This subgroup outcome underscores the durability of the response in deep responders, reaffirming the critical prognostic weight of CR rates [27].

Response rates improved between 30 and 90 days post-infusion (ORR30: 40.9%; ORR90: 57.1%), highlighting the importance of longitudinal assessment to capture delayed responses, particularly relevant in patients treated with Tisa-cel, where delayed activity is more common due to different T-cell kinetics [28].

While bridging therapy is often necessary in patients with aggressive disease, our findings suggest it negatively impacts survival. Patients not receiving bridging therapy showed superior PFS and OS compared to those who did, although this may reflect baseline disease biology rather than treatment effect. Bridging therapy itself did not affect CR rates but was associated with shorter OS (median: 9.1 months vs. not reached; *p* = 0.06), warranting further investigation of optimal patient selection and treatment timing. Indeed, this association did not hold in multivariate analysis, suggesting that other confounding factors, such as higher disease burden or more aggressive disease requiring bridging therapy, played a relevant role [29].

In our study, the median vein-to-vein time (V2Vt) was comparable to that reported in major clinical trials such as ZUMA-1 and ZUMA-7 (24 and 29 days, respectively), as well as in real-world data from larger referral center such as the Center for International Blood and Marrow Transplant Research (CIBMTR) USA registry (27 days) [25,26]. This finding underscores the effectiveness of streamlined logistics, close interdisciplinary collaboration, and proactive patient management of our center and highlights the critical role of the entire “brain-to-vein” pathway, from early identification and referral of eligible patients to the coordination of apheresis, manufacturing, and timely infusion. Ensuring efficiency across this continuum is essential for maximizing the benefits of CAR-T therapy, especially in aggressive and rapidly progressing diseases such as R/R DLBCL [30].

Axi-cel demonstrated numerically superior outcomes compared to Tisa-cel in terms of ORR90 (68.4% vs. 33.3%) and median PFS (not reached vs. 3 months; *p* = 0.04), though statistical significance was not consistently reached across all endpoints. These findings align with recent registry data, suggesting a more robust early expansion and response with Axi-cel, albeit with a slightly higher toxicity burden [31,32]. Nonetheless, the higher mortality in the Tisa-cel group (78.5% vs. 29.6%) is concerning and may reflect more advanced disease or patient frailty, particularly as Tisa-cel was only used in later lines of therapy in our cohort. At the same time, it is important to note that the worse outcomes observed in our cohort with Tisa-cel, which significantly impacted overall OS and PFS reported in this study, could partly reflect a bias of selection due its status as the first approved CAR-T product for DLBCL, which may have been caused by the a steeper learning curve in the management of a new therapy, especially during the critical early phase of the COVID-19 pandemic. Axi-cel use in the second-line setting appears to be associated with improved outcomes, echoing findings from ZUMA-7. Patients treated in the second line had numerically superior PFS and OS, although these were not statistically significant due to small numbers of patients included. These results support the earlier integration of CAR-T in the treatment algorithm for selected patients.

Upon univariate analysis, early response to therapy (at both 30 and 90 days) was strongly associated with improved outcomes, confirming its value as a surrogate marker for survival. The strong association of ORR at 30 and 90 days with better prognosis confirms that early deep response assessment can be a valuable indicator for predicting long-term benefit [25,33,34].

Conversely, bulky disease and multiple extranodal sites negatively impacted outcome, consistent with existing prognostic models [35]. However, in multivariate analysis, no single variable retained statistical significance, possibly due to sample size limitations, reinforcing the multifactorial nature of treatment outcomes in CAR-T recipients.

The safety profile of CAR-T in our real-world setting was generally manageable and in line with clinical trial reports [36]. CRS occurred in 81.4% of patients (93.1% in ZUMA-1, 87% in ZUMA-7, 58% in JULIET trials), mostly grade 1–2, with a low incidence of high-grade events (2.7%). ICANS was observed in 26.8% of patients (64% in ZUMA-1, 50% in ZUMA-7, 21% in JULIET trials), with three patients (8.1%) experiencing grade ≥ 3 events. Importantly, grade ≥ 3 toxicities occurred exclusively in the Axi-cel group, consistent with prior observations. The single case of macrophage activation syndrome and three COVID-19-related deaths reflect the need for supportive care, especially in immunocompromised patients during periods of high infectious risk.

Prolonged cytopenia were seen in nearly a quarter of patients on day 30, highlighting the ongoing need for monitoring and supportive care beyond the acute infusion period [37].

This study is limited by its single-center, retrospective design and relatively short follow-up, which may underestimate long-term survival benefits and late toxicities. The sample size also limits the power for subgroup analyses and multivariable modeling. Furthermore, heterogeneity in bridging therapies and patient selection (e.g., tFL, tMZL) may introduce confounding factors not fully accounted for.

Despite these limitations, this study offers valuable insights into CAR-T outcomes in R/R DLBCL, representing, to the best of our knowledge, the first Italian report that also includes the observation of second-line-treated patients. Importantly, our data highlight the feasibility of complex therapy such as CAR-T in a southern Italy region (Calabria) historically affected by logistical healthcare issues (patient accessibility and economic sustainability), which cause high levels extra-regional migration for medical treatment.

## 5. Conclusions

In this real-world study, CD19 CAR-T-cell therapy in patients with R/R DLBCL resulted in meaningful clinical benefits, particularly in those achieving CR and receiving Axi-cel in earlier treatment lines. The treatment was associated with an acceptable safety profile, with toxicities being generally manageable. Bridging therapy and high tumor burden emerged as potential negative prognostic factors. These data support the expanding the role of CAR-T in DLBCL and highlight the importance of early identification of candidates most likely to benefit from this treatment; the pretreatment T-cell-related biology measured in the tumor microenvironment should also be incorporated as part of inclusion criteria [38,39,40].

However, the geographic distribution of CAR-T-authorized centers in northern and central Italy, along with limited infrastructure, shortage of specialized staff, and fragmented referral systems may contribute to the southern centers’ persistent disadvantages in the future. Therefore, a better hub-and-spoke networks, streamlined referrals with digital coordination, standardized funding frameworks, and decentralized manufacturing CAR T platforms are required to overcome these challenges and improve outcomes for patients with DLBCL in southern Italy. These logistical aspects should receive greater attention, as the second-line use of CAR-T is rising with recent approval of Liso-cel [41] (Supplementary Table S1) and as new manufacturing and therapy models (e.g., allogeneic CAR T) [42] continue to evolve.

Furthermore, prospective studies are warranted to optimize patient selection, timing, and supportive care strategies in real-world settings, especially considering the data from the use of CAR-T in the front line (ZUMA-23, NCT05605899). Continued real-world data collection and analysis are crucial to optimize patient selection, refine management strategies, and further improve outcomes in this challenging patient population.

## Figures and Tables

**Table 1:** Baseline characteristics of patients included in this study.

Demographic and Clinical Characteristics	*n*	%
Sex		
Female	9	22
male	32	78
Age		
Median, y	66	
<70 y	34	82.9
>70 y	7	17.1
Performance Status (ECOG)		
0–1	39	4.9
>1	2	95.1
Disease involvement at relapse		
Bulky disease	17	56.7
Bone marrow involvement	4	9.8
>1 extranodal sites	4	9.8
Disease type		
Primary DLBCL	34	82.9
Transformed DLBCL	7	17.1
Status of disease before apheresis		
Controlled disease	20	52.2
Active disease	21	48.8
Prior stem-cell transplant	10	24.4
Number previous line of therapy		
1	10	24.4
2	23	56.1
3	5	12.2
4	3	7.3
Primary refractory disease	25	61
Bridging therapy		
Immuno-chemo	19	48.7
Chemo	8	19.5
IMiDs	1	2.5
RT	3	7.3
Steroids	3	7.3
None	7	17.1
CAR-T product infused		
Axi-cel	27	65.8
Tisa-cel	14	34.2
Line of treatment		
Second line	10	37
From third line	17	63

**Table 2:** Subgroup analysis of response rate (ORR90).

Variables	%	*p*-Value
Diagnosis		0.06
De novo	65.2	
Transformed	20	
Sex		0.144
Male	83.3	
Female	50	
CAR-T product		0.08
Axi-cel	68.4	
Tisa-cel	33.3	
Age		0.161
<70 y	62.5	
>70 y	25.5	
Performance status (ECOG)		0.240
0–1	59.2	
>1	0	
Disease involvement at relapse		
Bulky		0.466
Yes	70	
No	54.5	
Extranodal sites		0.376
0	60	
>1	33.3	
Line of therapy (LOT)		0.277
II line	50	
from III line	75	
Bridging therapy		0.755
Immuno-chemo	50	
None	58.3	

**Table 3:** Univariable analysis for OS.

Variables	*p*-Value	HR	95% CI	
			Lower	Upper
Sex				
Male	0.121	0.311	0.071	1.359
Age	0.312	0.982	0.949	1.017
<40 years	0.309	1.896	0.419	8.577
40–49 years	0.052	4.749	1.251	18.031
50–59 years	0.378	0.570	0.164	1.987
60–69 years	0.761	0.870	0.347	2.181
>70 years	0.659	0.756	0.218	0.768
Tumor bulk	0.044	3.322	1.032	10.711
>1 Extranodal sites	0.031	3.462	1.123	10.674
Transformed	0.374	1.608	0.565	4.678
ECOG > 1	0.572	1.432	3.422	6.919
Treatment				
Bridging therapy	0.096	5.578	0.738	42.134
Second line	0.838	0.854	0.186	3.910
ORR30	0.005	0.053	0.007	0.419
ORR90	0.011	0.125	0.025	0.653
Baseline high CRP (>3 mg/dL)	0.462	0.682	0.246	1.893
Baseline high ferritine (>650 ng/mL)	0.370	0.647	0.250	1.675

HR = hazard ratio; CI = confidence interval.

**Table 4:** Safety analysis of CAR-T-cell treatment.

Safety Analysis	*n*	%
Cytokine release syndrome/CRS		
Grade 1	29	78.5
Grade 2	5	13.5
Grade ≥ 3	1	2.7
Neurotoxicity/ICANS		
Grade 1	5	13.5
Grade 2	2	5.4
Grade ≥ 3	3	8.1
ICU admission < 30 days post-infusion	0	0
Therapy given for CRS/ICANS treatment		
Paracetamol	27	77.1
Corticosteroids	2	4.8
Tocilizumab	23	56

## Data Availability

All data relevant to this study are included in this article.

## Supplementary Materials

The following supporting information can be downloaded at https://www.mdpi.com/article/10.3390/cancers17172796/s1, Figure S1: OS and PFS by line of treatment: Kaplan–Meier curve for OS and PFS based on line of treatment with CAR-T; Table S1: Current CAR-T Italian commercial indications for DLBCL; Table S2: Multivariable analysis for OS; Table S3: Univariate analysis for CRS; Table S4: Univariate analysis for ICANS.

## Abbreviations

The following abbreviations are used in this manuscript:

DLBCL	Diffuse Large B-cell Lymphoma
CAR-T	Chimeric Antigen Receptor T cell
ORR	Overall Response Rate
PFS	Progression-Free Survival
OS	Overall Survival
CRS	Cytokine Release Syndrome
ICANS	Immune-Cell-Associated Neurotoxicity
ECOG	Eastern Cooperative Oncology Group
AIFA	Agenzia Italiana del Farmaco
