# Supplementary material for: CD19 CAR-T Outcomes in Patients with Relapsed/Refractory Diffuse Large B-Cell Lymphoma: A Retrospective Cohort Study from the Calabria Referral Center in Southern Italy
Source: Cancers (Basel). 2025 Aug 27;17(17):2796. doi: 10.3390/cancers17172796 (PMC12427354; doi:10.3390/cancers17172796)
Supplement: Supplementary file 1 [file cancers-17-02796-s001.zip › cancers-3798096-supplementary.pptx]

## Slide 1
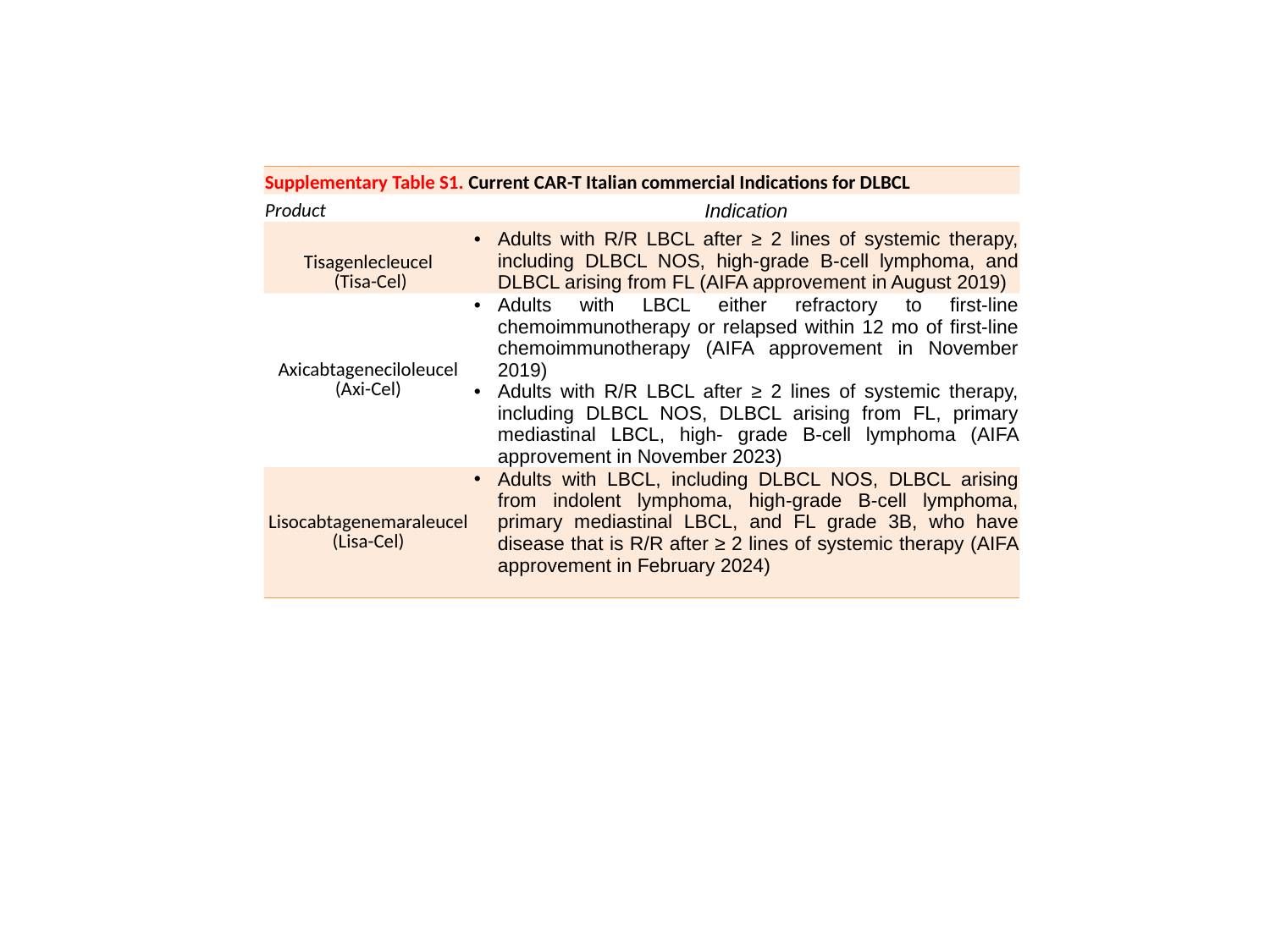

| Supplementary Table S1. Current CAR-T Italian commercial Indications for DLBCL | |
| --- | --- |
| Product | Indication |
| Tisagenlecleucel (Tisa-Cel) | Adults with R/R LBCL after ≥ 2 lines of systemic therapy, including DLBCL NOS, high-grade B-cell lymphoma, and DLBCL arising from FL (AIFA approvement in August 2019) |
| Axicabtageneciloleucel (Axi-Cel) | Adults with LBCL either refractory to first-line chemoimmunotherapy or relapsed within 12 mo of first-line chemoimmunotherapy (AIFA approvement in November 2019) Adults with R/R LBCL after ≥ 2 lines of systemic therapy, including DLBCL NOS, DLBCL arising from FL, primary mediastinal LBCL, high- grade B-cell lymphoma (AIFA approvement in November 2023) |
| Lisocabtagenemaraleucel (Lisa-Cel) | Adults with LBCL, including DLBCL NOS, DLBCL arising from indolent lymphoma, high-grade B-cell lymphoma, primary mediastinal LBCL, and FL grade 3B, who have disease that is R/R after ≥ 2 lines of systemic therapy (AIFA approvement in February 2024) |

## Slide 2
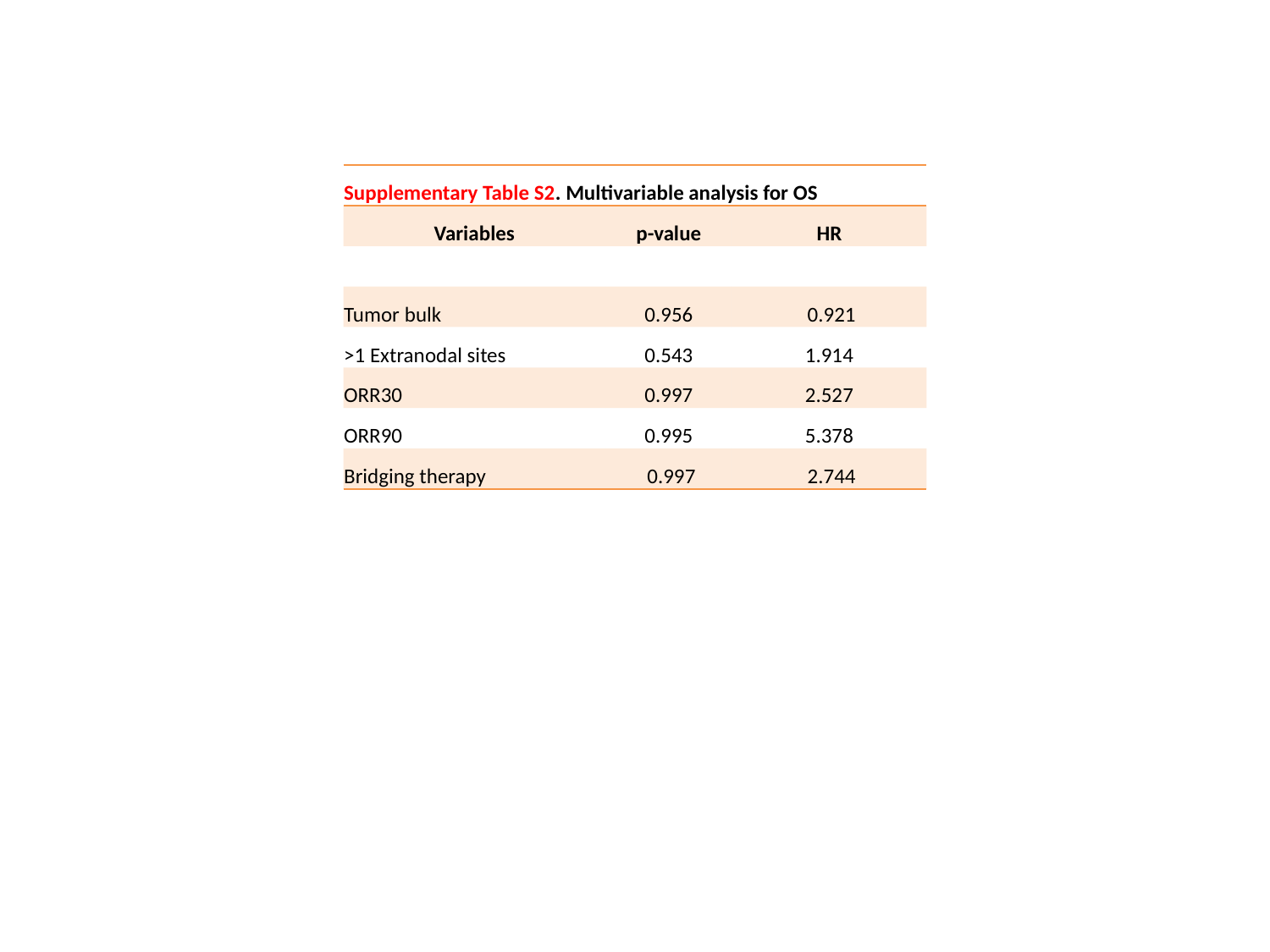

| Supplementary Table S2. Multivariable analysis for OS | | |
| --- | --- | --- |
| Variables | p-value | HR |
| | | |
| Tumor bulk | 0.956 | 0.921 |
| >1 Extranodal sites | 0.543 | 1.914 |
| ORR30 | 0.997 | 2.527 |
| ORR90 | 0.995 | 5.378 |
| Bridging therapy | 0.997 | 2.744 |

## Slide 3
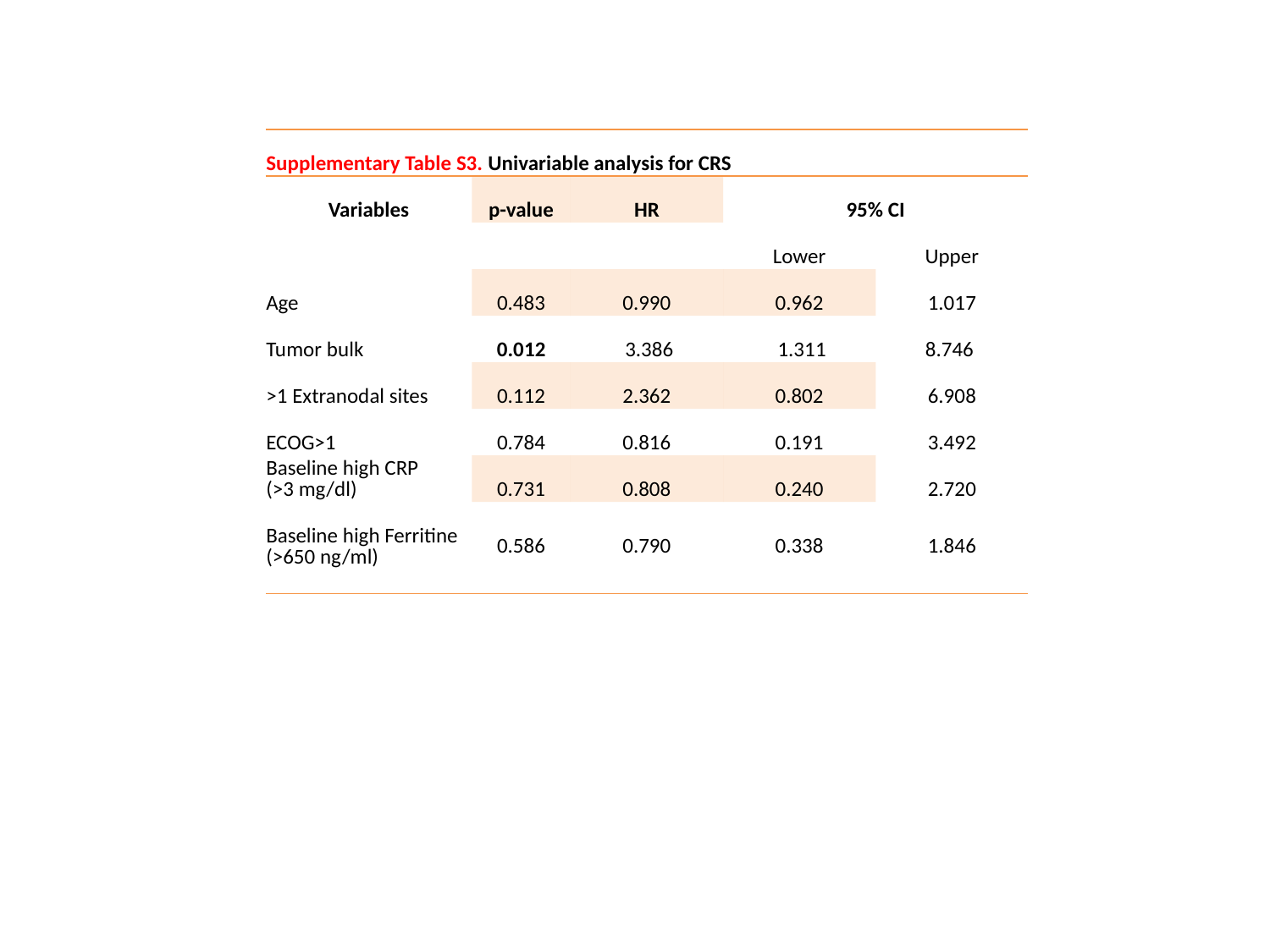

| Supplementary Table S3. Univariable analysis for CRS | | | | |
| --- | --- | --- | --- | --- |
| Variables | p-value | HR | 95% CI | |
| | | | Lower | Upper |
| Age | 0.483 | 0.990 | 0.962 | 1.017 |
| Tumor bulk | 0.012 | 3.386 | 1.311 | 8.746 |
| >1 Extranodal sites | 0.112 | 2.362 | 0.802 | 6.908 |
| ECOG>1 | 0.784 | 0.816 | 0.191 | 3.492 |
| Baseline high CRP (>3 mg/dl) | 0.731 | 0.808 | 0.240 | 2.720 |
| Baseline high Ferritine (>650 ng/ml) | 0.586 | 0.790 | 0.338 | 1.846 |

## Slide 4
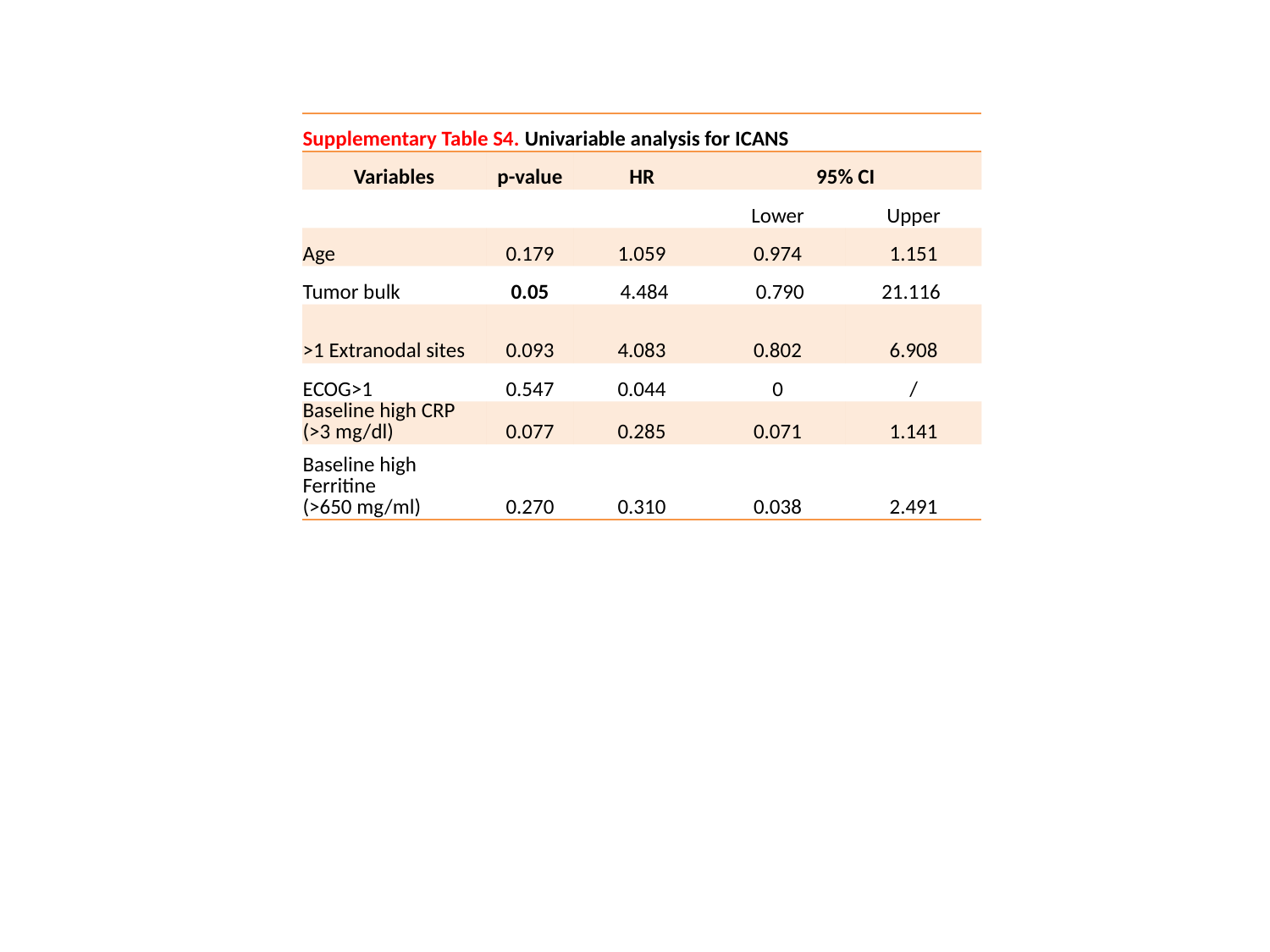

| Supplementary Table S4. Univariable analysis for ICANS | | | | |
| --- | --- | --- | --- | --- |
| Variables | p-value | HR | 95% CI | |
| | | | Lower | Upper |
| Age | 0.179 | 1.059 | 0.974 | 1.151 |
| Tumor bulk | 0.05 | 4.484 | 0.790 | 21.116 |
| >1 Extranodal sites | 0.093 | 4.083 | 0.802 | 6.908 |
| ECOG>1 | 0.547 | 0.044 | 0 | / |
| Baseline high CRP (>3 mg/dl) | 0.077 | 0.285 | 0.071 | 1.141 |
| Baseline high Ferritine (>650 mg/ml) | 0.270 | 0.310 | 0.038 | 2.491 |

## Slide 5
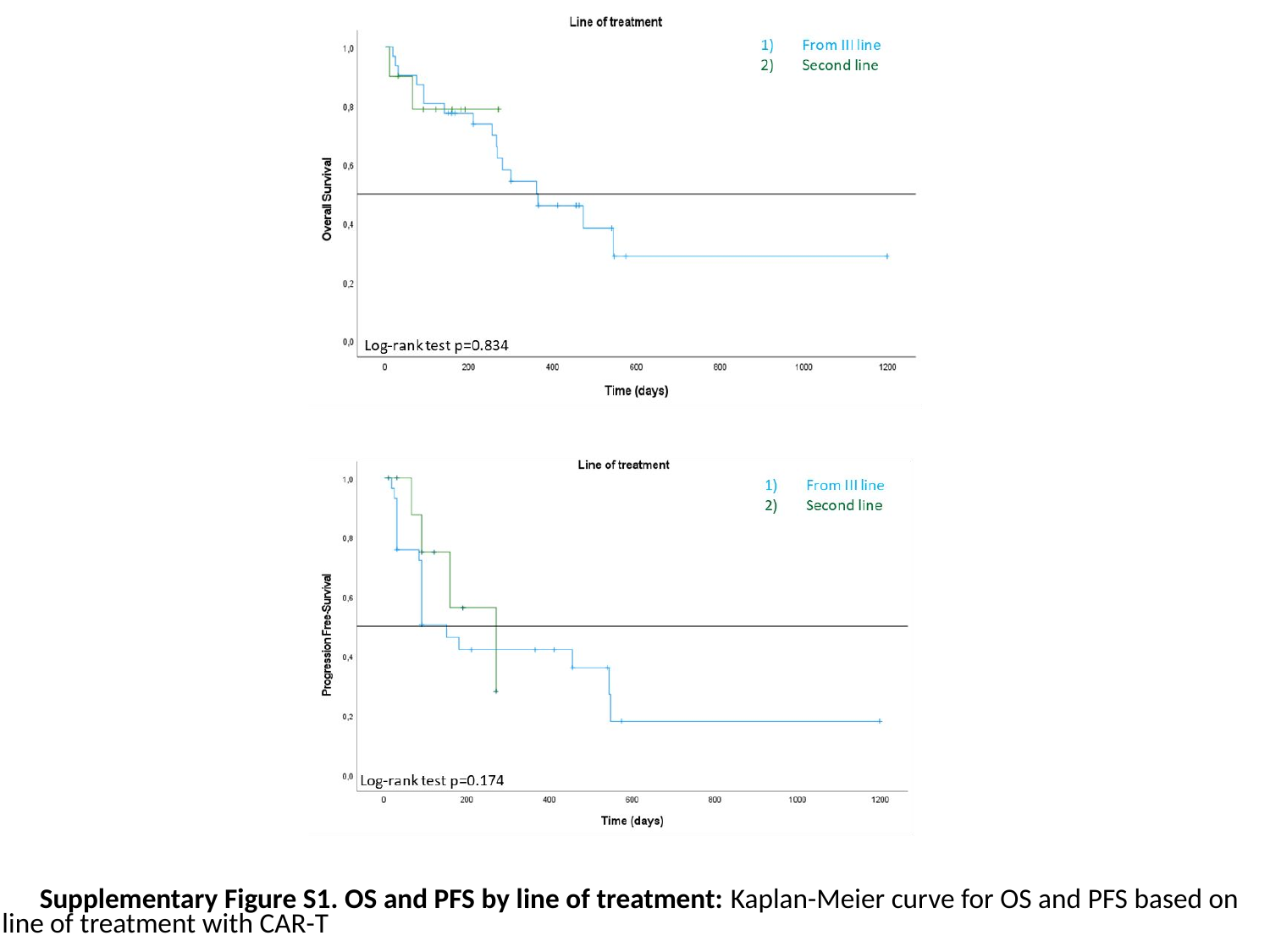

Supplementary Figure S1. OS and PFS by line of treatment: Kaplan-Meier curve for OS and PFS based on line of treatment with CAR-T
